# The Impact of Culture Variables on a 3D Human In Vitro Bone Remodeling Model: A Design of Experiments Approach

**DOI:** 10.1002/adhm.202301205

**Published:** 2023-07-16

**Authors:** Bregje W. M. de Wildt, Lizzy A. B. Cuypers, Esther E. A. Cramer, Annelieke S. Wentzel, Keita Ito, Sandra Hofmann

**Affiliations:** ^1^ Orthopaedic Biomechanics and Institute for Complex Molecular Systems (ICMS) Department of Biomedical Engineering Eindhoven University of Technology P.O. Box 513 Eindhoven 5600 MB The Netherlands; ^2^ Department of Regenerative Biomaterials Radboud Institute for Molecular Life Sciences Radboud University Medical Center P.O. Box 9101 Nijmegen 6525 GA The Netherlands

**Keywords:** 3D, bone remodeling, coculture, design of experiments, in vitro model

## Abstract

Human in vitro bone remodeling models, using osteoclast–osteoblast cocultures, can facilitate the investigation of human bone remodeling while reducing the need for animal experiments. Although current in vitro osteoclast–osteoblast cocultures have improved the understanding of bone remodeling, it is still unknown which culture conditions support both cell types. Therefore, in vitro bone remodeling models can benefit from a thorough evaluation of the impact of culture variables on bone turnover outcomes, with the aim to reach balanced osteoclast and osteoblast activity, mimicking healthy bone remodeling. Using a resolution III fractional factorial design, the main effects of commonly used culture variables on bone turnover markers in an in vitro human bone remodeling model are identified. This model is able to capture physiological quantitative resorption–formation coupling along all conditions. Culture conditions of two runs show promising results: conditions of one run can be used as a high bone turnover system and conditions of another run as a self‐regulating system as the addition of osteoclastic and osteogenic differentiation factors is not required for remodeling. The results generated with this in vitro model allow for better translation between in vitro studies and in vivo studies, toward improved preclinical bone remodeling drug development.

## Graphical abstract

Bones are continuously remodeled by bone‐forming osteoblasts and bone‐resorbing osteoclasts. A disruption in the resorption–formation balance can lead to pathologies like osteoporosis. This work presents a lab‐grown human bone model that is used to evaluate the impact of different cell culture methods on bone remodeling, thereby showing great potential for osteoporosis drug testing while reducing the need for animal experiments.

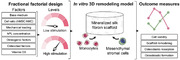

## 1 Introduction

Bone is a highly dynamic tissue continuously remodeled by bone resorbing osteoclasts, bone forming osteoblasts, and regulating osteocytes. Physiological bone remodeling involves resorption–formation coupling resulting in no net bone volume change. A shift in the remodeling balance, toward more resorption or formation, is a hallmark for bone pathologies like osteoporosis or osteopetrosis, respectively. Studies of these bone pathologies and their treatment testing are routinely performed in animal models. These animal models often represent human physiology insufficiently, which is likely one of the reasons why only 8%–10% of preclinically developed drugs are approved for regular clinical use.^[^ ^1^, ^2^, ^3^ ^]^ Human in vitro bone remodeling models could facilitate the investigation of human healthy and pathological bone remodeling while addressing the principle of reduction, refinement, and replacement of animal experiments (3Rs).^[^ ^4^, ^5^ ^]^

A coculture of osteoclasts and osteoblasts is minimally needed to mimic the bone remodeling process in vitro.^[^ ^6^ ^]^ For these cocultures, human monocytes (hMCs) and mesenchymal stromal cells (hMSCs) are most frequently used as progenitor cells which are differentiated in culture into osteoclasts and osteoblasts (and eventually osteocytes), respectively.^[^ ^7^ ^]^ To stimulate hMCs and hMSCs to undergo differentiation and subsequently study in vitro remodeling, a variety of culture conditions and outcome measures are used which differ for each research group and/or study aim.^[^ ^6^, ^7^ ^]^ Variations in culture protocols include, e.g., different cell ratios, different base media, the use of osteogenic/osteoclast supplements and their respective concentrations, and the application of mechanical load.^[^ ^7^ ^]^ These culture variables could lead to unequal stimulation of osteoblasts and osteoclasts which might induce a nonphysiological resorption–formation balance. In addition, highly concentrated medium supplements could interfere with resorption–formation coupling by overruling cell secreted remodeling regulating factors, including, e.g., receptor activator of nuclear factor κB ligand (RANKL) and macrophage colony‐stimulating factor (M‐CSF).^[^ ^4^, ^8^ ^]^ Thus, while current in vitro osteoclast–osteoblast cocultures have improved the mechanistic understanding of bone remodeling, common culture methods that equally support osteoclastic resorption and osteoblastic formation have not yet been identified. As such, they lack culture method standardization, hampering reproducibility and translatability between different in vitro models and potentially toward in vivo animal models and human data. In this regard, in vitro bone remodeling models could benefit from a thorough evaluation of the impact of culture variables on bone turnover, with the aim to find conditions that support resorption–formation coupling and thus physiological balanced remodeling.

**Figure 1:**
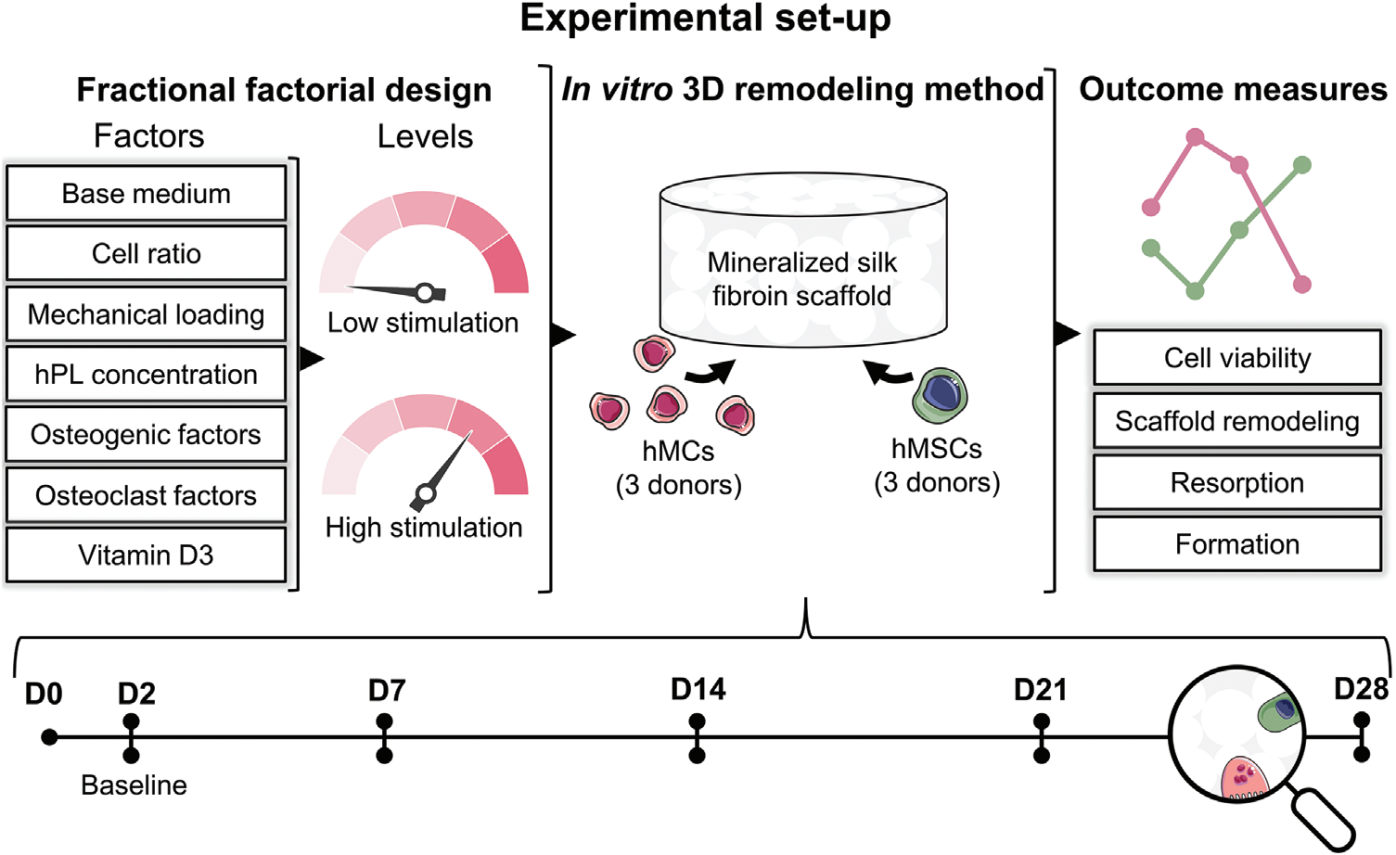
Experimental setup of the study. A fractional factorial design was used to study the influences of the culture variables (base medium, cell ratio, mechanical loading, hPL concentration, osteogenic differentiation factors, osteoclastic differentiation factors and 1,25‐dihydroxyvitamin D3) on cell viability, scaffold remodeling balance, osteoclastic resorption and osteoblastic formation in an in vitro bone remodeling model. A coculture of hMCs and hMSCs was maintained for a period of 28 days during which remodeling was tracked nondestructively. Abbreviations: human platelet lysate (hPL); human monocytes (hMCs); human mesenchymal stromal cells (hMSCs); day (D). Parts of the figure were modified from Biorender.com (https://biorender.com/, accessed on 6 October 2022) and Servier Medical Art, licensed under a Creative Common Attribution 3.0 Generic License (http://smart.servier.com/, accessed on 8 July 2021).

Researchers have already attempted to study the influences of culture variables on human osteoblast‐osteoclast cocultures. For example, studies looked at the influence of cell ratio on osteoclast formation,^[^ ^9^ ^]^ osteogenic factor addition and timing on osteogenic and osteoclastic differentiation,^[^ ^10^, ^11^ ^]^ and the replacement of the culture supplement fetal bovine serum (FBS) by serum free medium ^[^ ^12^ ^]^ or human platelet lysate (hPL) ^[^ ^13^ ^]^ on osteoclastic resorption. As such, most studies analyze the influence of only one culture variable on outcomes representing only aspects of bone remodeling. A specific combination of multiple variables, as well as functional outcomes on bone formation, resorption and their balance might lead to different results. A fractional factorial design of experiments (DoE) approach could facilitate the time‐efficient evaluation of the impact of multiple culture variables on in vitro remodeling outcomes.^[^ ^14^ ^]^ A fractional factorial design assumes that higher order interaction effects are insignificant. Factor main effects can therefore be evaluated using a fraction of the required experiments of a full factorial design study. While regularly used in most engineering fields, the DoE approach is relatively new for the bioengineering field. For bioengineering, DoE have been employed for, e.g., the optimization of biomaterials,^[^ ^15^, ^16^ ^]^ or the optimization of culture conditions for improved human pluripotent stem cell expansion,^[^ ^17^ ^]^ osteogenic differentiation of adipose derived hMSCs,^[^ ^18^ ^]^ or vascular network formation in bone‐like constructs.^[^ ^19^ ^]^ In this study, we used a fractional factorial design to evaluate the impact of culture variables on remodeling in a novel in vitro bone model (**Figure** **1**).^[^ ^20^ ^]^ The influence of commonly used culture variables,^[^ ^7^ ^]^ including base medium, cell ratio, mechanical loading, hPL concentration, osteogenic differentiation factors, osteoclast differentiation factors and 1,25‐dihydroxyvitamin D3, on mainly nondestructive and functional bone remodeling and cell viability outcomes was evaluated over a period of 28 days. By using this novel approach, we aimed at finding culture conditions that equally support osteoclastic and osteogenic differentiation of hMCs and hMSCs, respectively, followed by balanced in vitro remodeling.

As a result, we present an in vitro bone remodeling model that could capture quantitative resorption–formation coupling along all conditions. The model tended to be mostly influenced by mechanical loading and osteogenic differentiation factors; in vitro remodeling could be enhanced by the application of mechanical loading, while high stimulation with osteogenic factors disturbed the mineral resorption–formation coupling. Suitable conditions were found that could be applied to model high bone turnover or self‐regulating remodeling. In addition, the results generated with this in vitro model potentially allow for better translation between in vitro studies as well as to in vivo studies, toward improved preclinical bone remodeling drug development and a mechanistic understanding of human bone remodeling.

## 2 Results

### 2.1 Factor Selection and Experimental Matrix Creation

To select the parameters to be tested in the DoE setup, a database, as part of a systematic review, with culture conditions of all identified in vitro bone remodeling models was consulted.^[^ ^7^ ^]^ From this database, the following culture variables were identified: culture substrate/material, cell type, seeding density, base medium, coculture cell ratio, biomechanical environment, serum supplement, osteogenic differentiation factors (i.e., dexamethasone, β‐glycerophosphate and ascorbic acid), osteoclast differentiation factors (i.e., RANKL and M‐CSF), and the use of additional factors of which 1,25‐dihydroxyvitamin D3 was most commonly used. For this study, the influence of culture substrate/material, cell type and the hMSC seeding density were not included as these factors form the base of our in vitro model that was used to study the effect of the other factors.^[^ ^20^ ^]^ For the other factors, two levels (i.e., low stimulation and high stimulation) were assigned (**Table** **1**). For base medium, levels were α‐MEM and DMEM as most commonly used coculture base media.^[^ ^7^ ^]^ For cell ratio, ratios of 1:2 and 1:5 (hMSCs:hMCs) were included, where hMSC cell density was kept constant. For mechanical loading, static and dynamic loading were included, using a custom made spinner flask bioreactor at 300 RPM to apply fluid shear stress.^[^ ^21^ ^]^ As a serum supplement, hPL was used at 5% and 10% concentration since the most commonly used FBS can inhibit osteoclast resorption.^[^ ^12^, ^13^ ^]^ For osteogenic supplements, ascorbic acid was used in all conditions as a requirement for collagen synthesis. Dexamethasone was added in a low concentration of 10 × 10^−9^ m, which is a commonly used concentration for cocultures and believed to be the physiological glucocorticoid concentration known to stimulate osteogenesis and osteoclastogenesis.^[^ ^22^, ^23^ ^]^ The other commonly used coculture dexamethasone concentration of 100 × 10^−9^ m was used for high stimulation.^[^ ^7^ ^]^ β‐glycerophosphate was only added in the high stimulation condition at the most commonly used concentration of 10 × 10^−3^ m.^[^ ^7^ ^]^ As the material used in this study contained hydroxyapatite, resorption was expected to release sufficient phosphate for osteogenic differentiation and mineralization to leave out β‐glycerophosphate in the low stimulation condition. M‐CSF and RANKL were only added in high stimulation conditions at commonly used concentrations of 50 ng mL^−1^.^[^ ^7^ ^]^ The concentration of 1,25‐dihydroxyvitamin D3 was set at the common concentration 10 × 10^−9^ m in high stimulation conditions, whereas in low stimulation conditions no 1,25‐dihydroxyvitamin D3 was added (**Table** **1**).

**Table 1:** Evaluated factors and their corresponding levels

	Factor		Level		Unit
			Low	High	
A	Base medium		α‐MEM	DMEM	–
B	Cell ratio		1:2	1:5	–
C	Mechanical loading^a)^		0	300	RPM
D	hPL concentration		5	10	%
E	Osteogenic factors^b)^	Dexamethasone	10	100	nm
		β‐Glycerophosphate	0	10	mm
F	Osteoclast factors	M‐CSF	0	50	ng mL^−1^
		RANKL^c)^	0	50	ng mL^−1^
G	1,25‐Dihydroxyvitamin D3		0	10	nm

Abbreviations: human platelet lysate (hPL); macrophage colony‐stimulating factor (M‐CSF); receptor activator of nuclear factor κB ligand (RANKL).

^a)^ Applied from day 2 in culture;

^b)^ Ascorbic acid was present in all cultures;

^c)^ Added from day 2 in culture.

For the resulting seven factors with two levels, a resolution III fractional factorial design was randomly created using R (version 4.1.2) ^[^ ^24^ ^]^ with the Rcmdr DoE plugin (version 0.12‐3, Ulrike Groemping),^[^ ^25^ ^]^ leading to eight experimental runs (**Table** **2**). Resolution ≥ III designs are considered appropriate for screening purposes. An additional run was included in which all factors had level low, which served as a negative control (run 9), a positive control (all factors level high) was already part of the design (run 7) (Table 2).

**Table 2:** Experimental matrix

Run	A	B	C	D	E	F	G
1	α‐MEM	1:5	Dynamic	5% hPL		OCL fact	
2	α‐MEM	1:5	Static	5% hPL	OG fact		vitD3
3	DMEM	1:5	Static	10% hPL			
4	α‐MEM	1:2	Dynamic	10% hPL			vitD3
5	DMEM	1:2	Static	5% hPL		OCL fact	vitD3
6	DMEM	1:2	Dynamic	5% hPL	OG fact		
7	DMEM	1:5	Dynamic	10% hPL	OG fact	OCL fact	vitD3
8	α‐MEM	1:2	Static	10% hPL	OG fact	OCL fact	
9	α‐MEM	1:2	Static	5% hPL			

Abbreviations: human platelet lysate (hPL); osteogenic differentiation factors (OG fact); osteoclast differentiation factors (OCL fact); 1,25‐dihydroxyvitamin D3 (vitD3). A: base medium; B: cell ratio; C: mechanical loading; D: human platelet lysate concentration; E: osteogenic factors; F: osteoclast factors; G: 1,25‐dihydroxyvitamin D3.

### 2.2 The Impact of Culture Variables on Cell Viability

**Figure 2:**
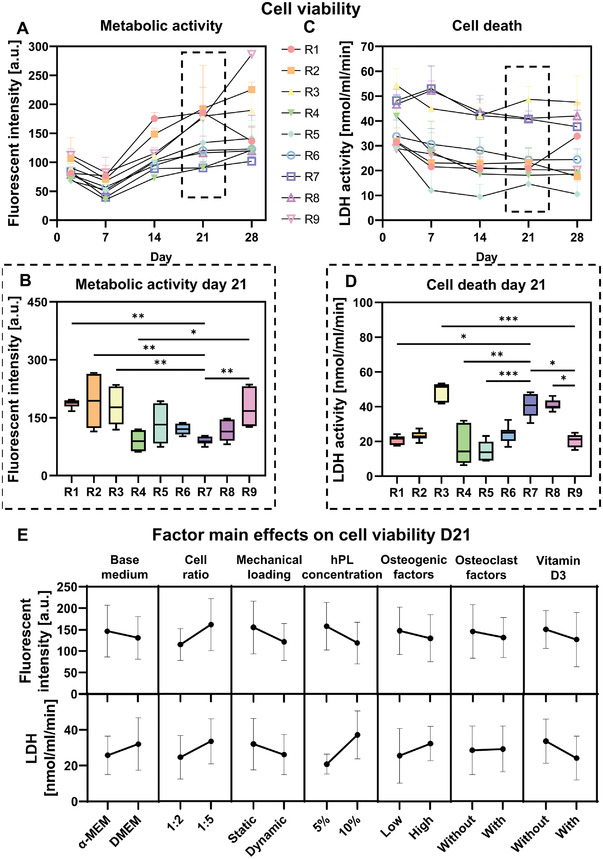
Cell viability testing of experimental runs. A) Metabolic activity measurements using PrestoBlue on days 2, 7, 14, 21, and 28. B) Day 21 metabolic activity measurements, *p* < 0.05 (Kruskal–Wallis and Dunn's post hoc tests). C) Cell death measured by LDH release in the medium on days 2, 7, 14, 21, and 28. D) Day 21 cell death measurements, *p* < 0.05 (Kruskal–Wallis and Dunn's post hoc tests). E) Factor main effects and standard deviations on day 21 cell viability outcome measures. None of the factors had a significant influence on cell viability outcomes. R7: positive control; R9: negative control (*p < 0.05, **p < 0.01, ***p < 0.001). Abbreviations: lactate dehydrogenase activity (LDH); run (R); human platelet lysate (hPL).

We first evaluated the influence of culture variables on cell viability. To measure cell metabolic activity, the conversion of resazurin to fluorescent resorufin by viable cells was measured. From day 7 to day 28, metabolic activity increased for all experimental runs (**Figure** **2**A). When comparing the metabolic activity on day 21 of each run with the metabolic activity of the positive (only high stimulation—run 7) and negative (only low stimulation—run 9) control, a statistically significant lower metabolic activity was found in the positive control (run 7) when compared with runs 1, 2, and 3 and the negative control (run 9) (Figure 2B). The negative control also had a statistically significant higher metabolic activity than run 4 (Figure 2B). Lactate dehydrogenase (LDH) activity in the supernatant, as a measure for cell death, initially decreased for most experimental runs while toward day 21 it tended to stabilize or increase slightly (Figure 2C). While the metabolic activity was relatively low in the positive control, a statistically significant higher day 21 LDH activity was found when comparing the positive control with runs 1, 4, and 5 and the negative control (run 9) (Figure 2D). Overall, cell death on day 21 was lowest in the negative control with a statistically significant difference when comparing to runs 3 and 8 (Figure 2D). Nevertheless, no differences in DNA content were observed between experimental runs after 28 days of culture (Figure S1, Supporting Information).

To study the influence of each culture variable and their corresponding level on metabolic activity and cell death, factor main effect plots were generated (Figure 2E). No significant contribution to metabolic activity nor cell death was found from one of the factors. The absence of significant factors indicates that a combination of multiple factors contributed to the found differences between the experimental runs. Nevertheless, from the metabolic activity main effect plots and normal effect plots, a high cell ratio (1:5) tended to positively influence metabolic activity (Figure 2E; Figure S2, Supporting Information). This is likely because of the higher total number of cells as the hMSC seeding density remained constant. Interestingly, all other factors tended to negatively impact metabolic activity when high stimulation was applied (Figure 2E; Figure S2, Supporting Information), which might be attributed to differences in energy metabolism of undifferentiated and differentiated progenitor cells.^[^ ^26^, ^27^, ^28^ ^]^ Moreover, a high concentration of hPL tended to increase LDH activity (Figure 2E; Figure S2, Supporting Information), likely caused by the presence of LDH in human platelets.^[^ ^29^ ^]^

### 2.3 The Impact of Culture Variables on Scaffold Remodeling

**Figure 3:**
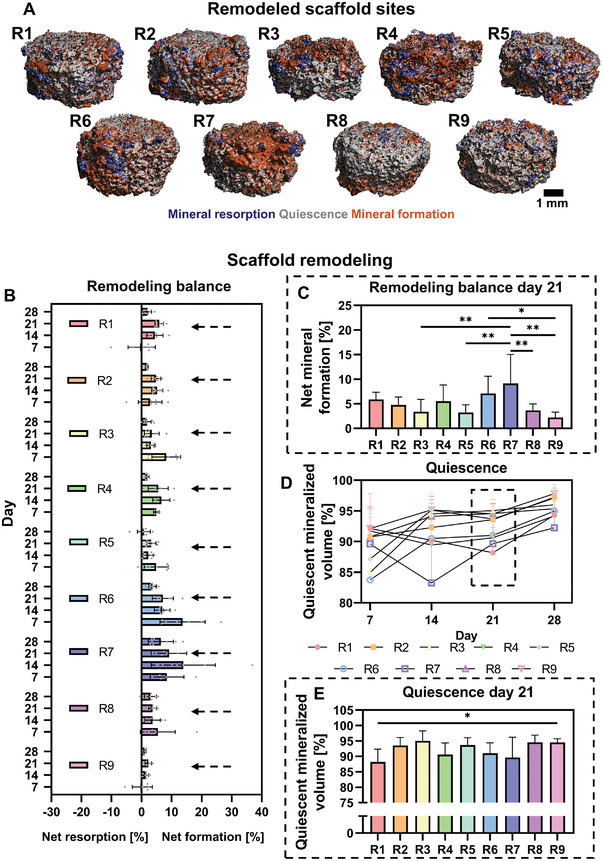
Scaffold remodeling of experimental runs. A) Remodeled scaffolds sites between days 2 and 28, obtained with µCT. B) µCT‐based formed mineral‐resorbed mineral as measure for remodeling balance of days 2–7, 7–14, 14–21, and 21–28. The dashed arrows represent the time points highlighted in (C). C) Days 14–21 remodeling balance, *p* < 0.05 (one‐way ANOVA and Holm–Šídák's post hoc tests). D) µCT‐based quiescent mineral or un‐remodeled scaffold mineral of days 2–7, 7–14, 14–21, and 21–28. E) Days 14–21 quiescent mineral, *p* < 0.05 (one‐way ANOVA and Holm–Šídák's post hoc tests). R7: positive control; R9: negative control (*p < 0.05, **p < 0.01). Abbreviation: run (R).

In our effort to reach balanced remodeling in vitro, the influences of culture variables on bone turnover parameters were evaluated. As such, mineral resorption, formation, and quiescence (un‐remodeled) sites were visualized after registration of day 28 microcomputed tomography (µCT) images to day 2 images, remodeling was observed in all experimental runs (**Figure** **3**A). While in the negative control (run 9) limited remodeling was observed, the positive control (run 7) showed extensive remodeling with mostly mineral formation (Figure 3A). Quantification of the percentage formed and resorbed mineral between days 2–7, 7–14, 14–21, and 21–28, allowed for calculating the balance between formed and resorbed mineral (i.e., mineral formation–mineral resorption) (Figure 3B). Remarkably, in all experimental runs more formation than resorption was observed for most time points (Figure 3B). Over time, the negative control (run 9) showed most balanced remodeling with limited net resorption or formation, while in the positive control (run 7) a relatively high net formation was observed at all time points (Figure 3B). From day 14 to day 21, there was indeed a significantly higher net formation in the positive control when compared to runs 3, 5, and 8, and the negative control (Figure 3C), which have in common that they were all cultured statically. In addition to the positive control, run 6 also had a significantly higher net formation than the negative control (Figure 3C). Comparison of the percentage quiescent scaffold mineralized volume revealed that most scaffold was remodeled in the initial 21 days, observed by a small increase in quiescent volume after day 21 consistent for all experimental runs (Figure 3D). Overall, most scaffold remodeling seemed to take place in the positive control and least remodeling in the negative control (Figure 3D). From day 14 to day 21, least quiescent scaffold mineralized volume was observed in run 1, with statistically significant less quiescent volume than the negative control (run 9) (Figure 3E).

### 2.4 The Impact of Culture Variables on Resorption

**Figure 4:**
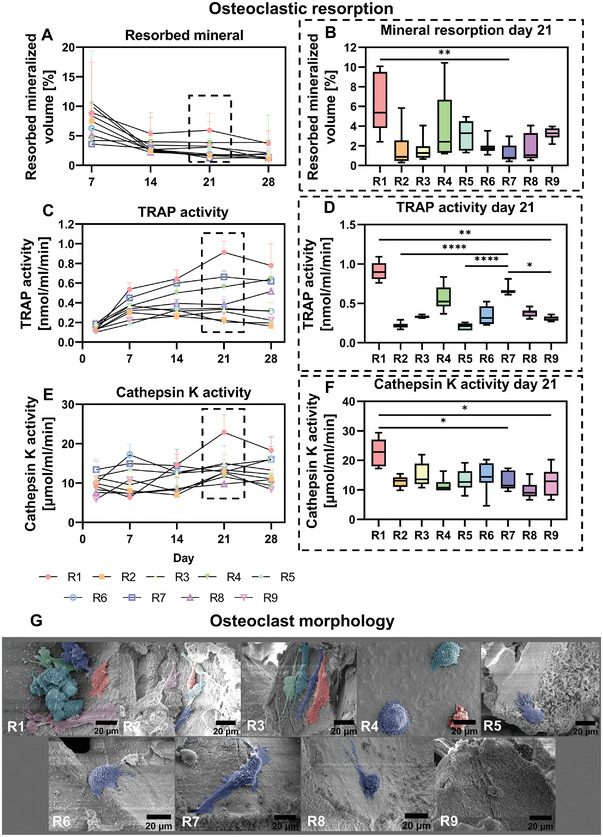
Osteoclastic resorption of experimental runs. A) µCT‐based resorbed mineral of days 2–7, 7–14, 14–21, and 21–28. B) Days 14–21 resorbed mineral, *p* < 0.05 (Kruskal–Wallis and Dunn's post hoc tests). C) TRAP activity in the medium on days 2, 7, 14, 21, and 28. D) Day 21 TRAP activity measurements, *p* < 0.05 (Kruskal–Wallis and Dunn's post hoc tests). E) Cathepsin K activity in the medium on days 2, 7, 14, 21, and 28. F) Day 21 cathepsin K activity measurements, *p* < 0.05 (Kruskal–Wallis and Dunn's post hoc tests). G) Visualization of osteoclast‐like cells on day 28 with SEM. Different colors represent different osteoclasts. R7: positive control; R9: negative control (*p < 0.05, **p < 0.01, ****p < 0.0001). Abbreviations: tartrate‐resistant acid phosphatase (TRAP); run (R); scanning election microscopy (SEM).

To study the contribution of osteoclastic resorption to the remodeling balance, we measured the two enzymatic osteoclast markers tartrate resistant acid phosphatase (TRAP) and Cathepsin K over time to support the µCT mineral resorption data. Over the culture period, most mineralized volume was resorbed in run 1 (**Figure** **4**A). In both the positive (run 7) and negative (run 9) controls, limited resorption was observed (Figure 4A). When comparing the resorbed mineralized volume from day 14 to day 21, statistically significant more mineral was resorbed in experimental run 1 when compared with the positive control (Figure 4B). Interestingly, the osteoclast factor‐lacking run 4 tended to have relatively high resorbed mineralized volume as well. For runs 1 and 4, the high resorbed mineralized volume was reflected in a relatively high TRAP activity and for run 1 also Cathepsin K activity (Figure 4C–F). Although limited resorbed mineralized volume was observed for the positive control (run 7), a relatively high TRAP activity was measured (Figure 4C). On day 21, a statistically significant higher TRAP activity was measured in runs 1 and 7, when compared to the negative control (run 9) (Figure 4D). When compared to the positive control (run 7), a statistically significant lower TRAP activity was found in runs 2 and 5 (Figure 4D). From scanning electron microscopy (SEM) images, osteoclast‐like cells were observed in run 1 – 8 (Figure 4G). No apparent osteoclast‐like cells (i.e., >10 µm in diameter and a ruffled boarder) were found in the negative control (run 9).

**Figure 5:**
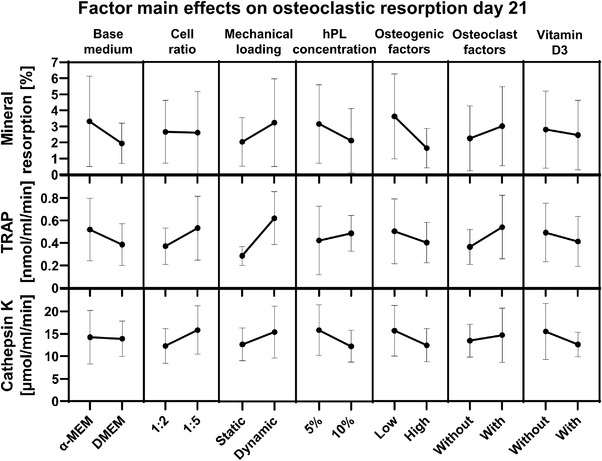
Factor main effects and standard deviations on day 21 osteoclastic resorption outcome measures. None of the factors had a significant influence on resorption outcomes. Abbreviations: tartrate‐resistant acid phosphatase (TRAP); human platelet lysate (hPL).

To study the influence of each culture variable and their corresponding level on mineral resorption, TRAP activity and cathepsin K activity, factor main effect plots were generated (**Figure** **5**). No statistically significant contribution to mineral resorption, TRAP activity or cathepsin K activity was found from one of the factors. From the main effect plots and normal effect plots, mechanical loading tended to positively influence mineral resorption and TRAP activity, while the addition of high concentrations of osteogenic supplements tended to negatively influence mineral resorption (Figure 5; Figure S3, Supporting Information).

### 2.5 The Impact of Culture Variables on Formation

**Figure 6:**
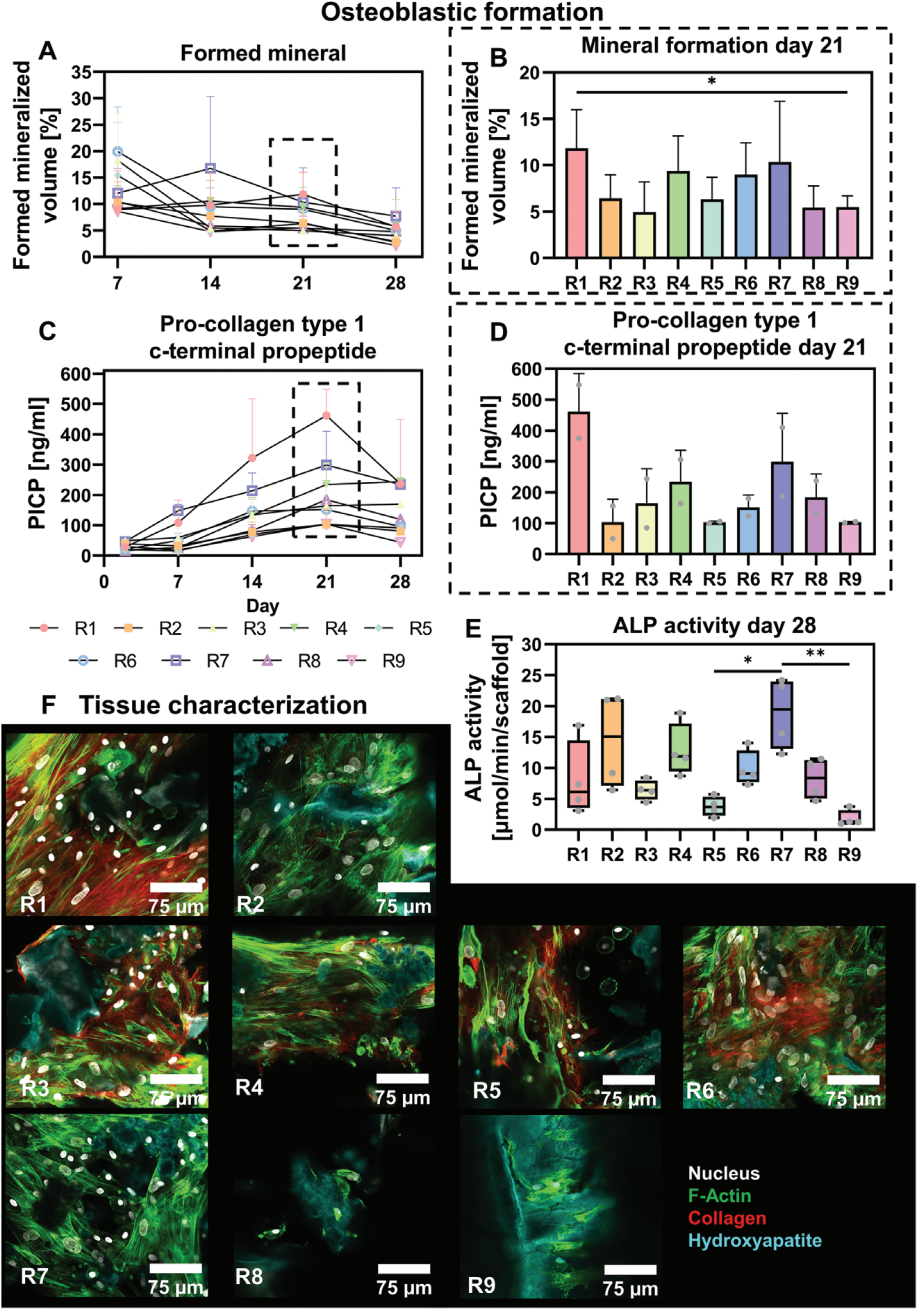
Osteoblastic formation of experimental runs. A) µCT‐based formed mineral of days 2–7, 7–14, 14–21, and 21–28. B) Days 14–21 formed mineral, *p* < 0.05 (one‐way ANOVA and Holm–Šídák's post hoc tests). C) PICP release in medium from days 2, 7, 14, 21, and 28. D) Day 21 PICP release measurements. E) ALP activity in cell lysates on day 28, *p* < 0.05 (Kruskal–Wallis and Dunn's post hoc tests). F) Visualization of bone‐like tissue in cultured constructs on day 28 with confocal microscopy, stained for collagen (red), F‐actin (green), hydroxyapatite (cyan), and the nucleus (gray). R7: positive control; R9: negative control (*p < 0.05, **p < 0.01). Abbreviations: procollagen 1 c‐terminal propeptide (PICP); alkaline phosphatase (ALP); run (R).

When evaluating formed mineralized volume over time, the positive control (run 7) tended to have consistently high mineral formation while in the negative control (run 9), a relatively low formed mineralized volume was observed (**Figure** **6**A). On day 21, only for run 1 a statistically significant higher formed mineralized volume was observed when compared to the negative control (Figure 6B). We also measured procollagen 1 c‐terminal propeptide (PICP) over time as a marker for collagen formation, as well as alkaline phosphates (ALP) on day 21 as an enzymatic marker for osteogenesis. Where most mineral formation was observed in runs 1 and 7, also highest collagen type 1 formation was observed in these conditions (Figure 6C,D). In the negative control, limited collagen type 1 formation was observed. ALP activity measurements on the cell lysate of day 28 revealed highest ALP activity in the positive control and lowest in the negative control (Figure 6E). For the positive control, a statistically significant higher ALP activity was observed when compared to the ALP activity of run 5 and the negative control. Visualization of the constructs with confocal microscopy demonstrated collagen formation in runs 1, 3, 4, 5, and 6 (Figure 6F). Remarkably, although relatively high PICP was observed in the positive control, almost no collagen was observed in the microscopy samples (Figure 6F). In all conditions, hydroxyapatite was mainly observed on the SF scaffold trabeculae rather than in the by the cells produced extracellular matrix (Figure 6F).

**Figure 7:**
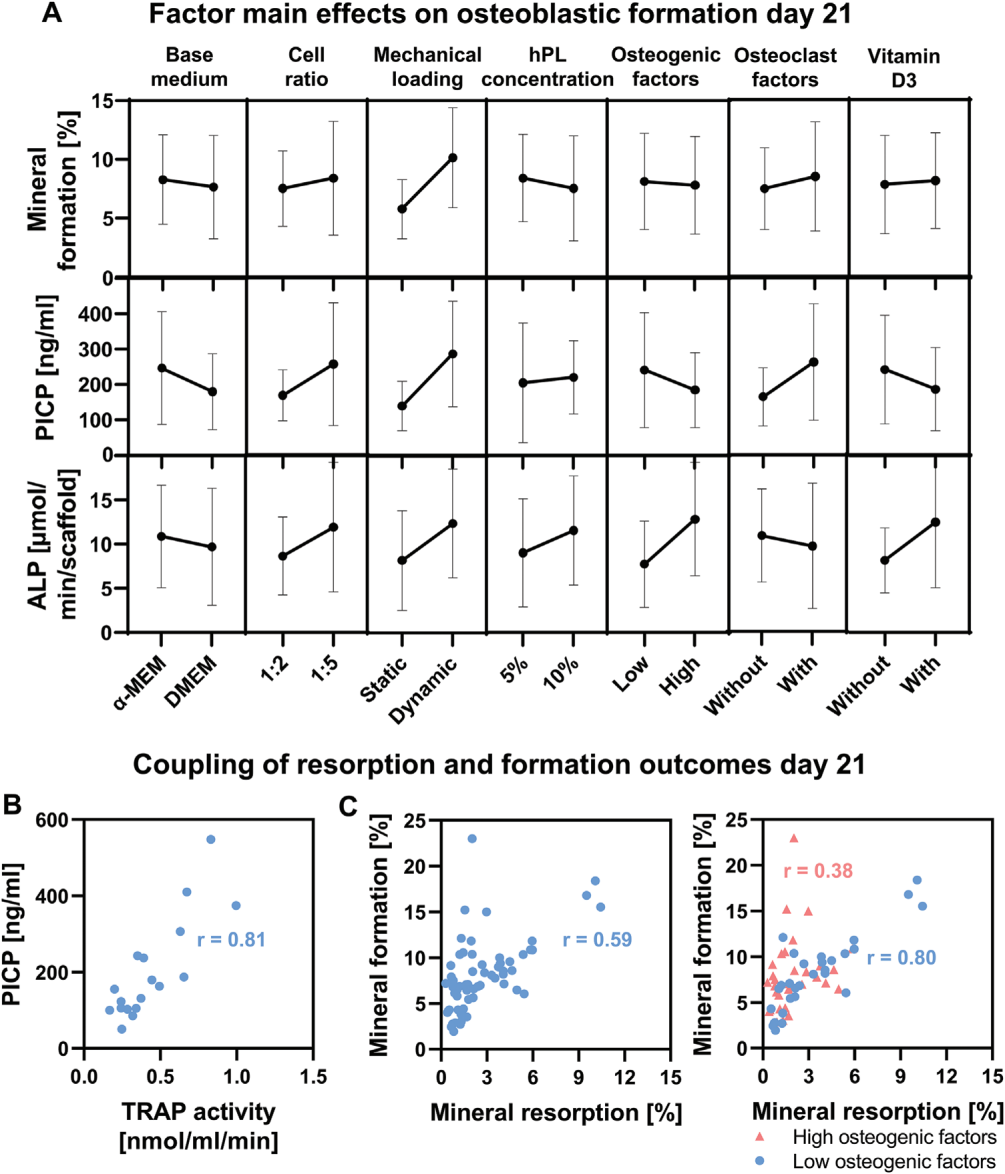
A) Factor main effects and standard deviations on day 21 osteoblastic formation outcome measures. None of the factors had a significant influence on formation outcomes. Correlation/coupling of resorption and formation outcomes for B) organic matrix resorption and formation (TRAP and PICP), and C) inorganic matrix resorption and formation, in the presence and absence of high stimulation with osteogenic differentiation factors. Abbreviations: human platelet lysate (hPL); tartrate‐resistant acid phosphatase (TRAP); procollagen 1 c‐terminal propeptide (PICP).

To study the influence of each culture variable and their corresponding level on mineral formation, collagen type 1 formation and ALP activity, factor main effect plots were generated (**Figure** **7**A). No significant contribution to mineral formation, collagen type 1 formation, and ALP activity was found from one of the factors. From the main effect plots and normal effect plots, mechanical loading tended to positively influence mineral formation and collagen type 1 formation (Figure 7A; Figure S3, Supporting Information). Other factors did not seem to influence mineral formation (Figure 7A; Figure S3, Supporting Information). ALP activity tended to be positively influenced by high concentrations of osteogenic supplements (Figure 7A; Figure S3, Supporting Information). As mineral formation was relatively high in groups where mineral resorption was elevated in the absence of high stimulation with osteogenic factors, we investigated the coupling between resorption and formation markers (Figure 7B,C). Interestingly, a strong positive correlation (*r* = 0.81, *p* < 0.0001) was observed between the osteoclastic resorption marker TRAP and the collagen formation marker PICP (Figure 7B). In line with these results, main effect plots and normal effect plots for TRAP activity and PICP concentration followed a similar pattern (Figures 5 and 7A; Figure S3, Supporting Information), which could suggest an influence of osteoclastic differentiation and/or TRAP activity on osteoblastic collagen formation. When investigating the coupling between mineral resorption and formation, a moderate positive correlation (*r* = 0.59, *p* < 0.0001) was found (Figure 7C). When splitting the data into high and low stimulation with osteogenic factors, a weak positive (*r* = 0.38, *p* < 0.05) correlation between mineral resorption and formation was found in highly stimulated scaffolds whereas a strong positive correlation (*r* = 0.80, *p* < 0.0001) was found when low stimulation was applied (Figure 7C). This indicates that resorption – formation coupling can be disturbed by high stimulation with osteogenic factors.

## 3 Discussion

Human in vitro bone remodeling models, using osteoclast–osteoblast cocultures, could facilitate the investigation of human physiological and pathological bone remodeling while addressing the principle of 3Rs for animal experiments.^[^ ^4^, ^5^ ^]^ Although current in vitro osteoclast–osteoblast cocultures have improved our understanding of bone remodeling, they lack standardization of culture methods and outcome measurements, hampering reproducibility and translatability to in vivo animal models and in vivo human data. In this regard, in vitro bone remodeling models could benefit from a thorough evaluation of the impact of culture variables on bone turnover, with the aim to reach balanced osteoclast and osteoblast activity mimicking physiological bone remodeling. Using a resolution III fractional factorial design, we identified the main effects of commonly used culture variables at high and low stimulation on bone remodeling and differentiation markers in an in vitro human bone remodeling model.

We first evaluated the influence of culture variables on cell viability. Metabolic activity tended to decrease with high stimulation of all factors other than cell ratio. The positive influence of cell ratio on metabolic activity is likely caused by a higher total number of cells at the start of the experiment. By only increasing the hMC density and maintaining the hMSC density over the two different cell ratios, the influence of cell ratio on remodeling outcomes was only affected by a change in density of hMCs instead of both cell types and their potential interactions. The negative influence on metabolic activity of the other culture variables with high stimulation might be explained by differences in energy metabolism of undifferentiated and differentiated progenitor cells. In this study, metabolic activity was measured by the reduction of resazurin to fluorescent resorufin by aerobic respiration. In contrast to our metabolic activity results indicating less metabolic activity upon cell differentiation, osteogenic differentiation of hMSCs has shown to increase the portion of aerobic respiration to the cells’ energy metabolism.^[^ ^26^ ^]^ Moreover, osteoclast differentiation is associated with increased mitochondrial biosynthesis and oxygen consumption rate, likely enhancing aerobic respiration.^[^ ^27^, ^28^ ^]^ Thus, osteogenic and osteoclastic differentiation were expected to increase metabolic activity. Although high stimulation did not always lead to improved cell differentiation as measured with enzymatic activity markers (i.e., ALP, TRAP, and Cathepsin K), one hypothesis for this contradiction might be that limited exogenous factor stimulation enhances endogenous factor production. To confirm this hypothesis, further cell secretome quantification using for example multiplex ELISA is required. If cells with limited stimulation are indeed producing more endogenous factors, limited stimulation might be essential to create self‐regulating models.

In our effort to reach balanced in vitro remodeling, the impact of culture variables on bone turnover was evaluated. Although the negative control showed most balanced resorption and formation, only limited remodeling could be detected, while the ability to capture remodeling is imperative for in vitro remodeling models. In contrast, the positive control showed least balanced resorption and formation, with a relatively high net formation and low volume of quiescent mineral. As such, both low and high stimulation are nonoptimal to mimic bone homeostasis in vitro. With the least quiescent mineral, experimental run 1 (α‐MEM, 1:5, dynamic, 5% hPL, and osteoclast factors) stood out. When evaluating the resorption dynamics of this experimental run, most mineral resorption and highest activity of osteoclast enzymatic markers TRAP and Cathepsin K were measured, and typical osteoclast‐like cells were identified. Therefore, run 1 is considered optimal for in vitro bone resorption. Remarkably, in terms of osteoblastic formation, run 1 also showed most mineral and collagen type 1 formation. Only for ALP activity in the cell lysates, run 1 showed levels in between the positive and negative control. This appears contradictory but might well be due to downregulation of ALP activity upon osteoblast maturation.^[^ ^30^ ^]^

Thus far, the use of exogenous osteoclast differentiation factors (i.e., RANKL and M‐CSF) in osteoclast–osteoblast cocultures is generally considered crucial for the development of functional osteoclasts,^[^ ^4^ ^]^ even though exogenous application of these factors could overrule the natural RANKL/osteoprotegerin (OPG) ratio as important regulator in physiological and pathological bone remodeling.^[^ ^4^, ^31^ ^]^ Strikingly, when we replaced osteoclastogenic and osteogenic differentiation factors by 1,25‐dihydroxyvitamin D3 (run 4; α‐MEM, 1:2, dynamic, 10% hPL, and 1,25‐dihydroxyvitamin D3), we observed relatively high osteoclast and osteoblast activity. Even in the absence of differentiation factors, relatively high levels of mineral resorption, TRAP activity, mineral formation, collagen type 1 production, and ALP activity were found. When no differentiation factors were used in human peripheral blood mononuclear cell (PBMC)‐osteoblast cocultures, no osteoclastic differentiation of PBMCs was observed.^[^ ^32^ ^]^ Other researchers found that when human PBMCs and hMSCs were cocultured on osteoblast derived matrix in the absence of osteogenic and osteoclastic differentiation factors, resorption was comparable to PBMC mono‐cultures treated with M‐CSF and RANKL.^[^ ^33^ ^]^ Moreover, before the discovery of RANKL and the ability to clone this factor, stromal cells and osteoblasts were used as a tool for osteoclastic differentiation in vitro.^[^ ^34^ ^]^ We therefore believe that osteoclastic differentiation in osteoclast–osteoblast coculture in the absence of exogenous RANKL is possible under the correct circumstances. With the use of 1,25‐dihydroxyvitamin D3 in the absence of differentiation factors in experimental run 4, RANKL expression by hMSCs/osteoblasts and subsequent osteoclastic differentiation might have been stimulated as earlier demonstrated.^[^ ^34^, ^35^ ^]^ It would be interesting to quantify M‐CSF, RANKL and OPG produced in the experimental runs to investigate the influence of 1,25‐dihydroxyvitamin D3 on the osteoclastic differentiation potential in our cocultures, with the ambition to circumvent the use of exogenous osteoclastic differentiation factors in future. Nevertheless, it could well be that osteogenic and osteoclastogenic differentiation were stimulated by the combination of factors in run 4 including mechanical loading and a high concentration of hPL.^[^ ^13^, ^36^ ^]^

The trend that resorption and formation were both similarly enhanced in, e.g., experimental runs 1 and 4 raised the expectation that resorption and formation were coupled in our in vitro model. Indeed, resorption and formation were correlated for both mineral resorption/formation and organic matrix resorption/formation. By adding an exogeneous phosphate source to the model (β‐glycerophosphate), mineral resorption–formation coupling was disturbed. Most likely, osteoclasts released sufficient calcium phosphate from the mineralized scaffold for subsequent formation. In vivo, coupling includes communication through secreted and cell‐bound factors, topographical cues, and the release of growth factors from the bone matrix, with the main goal to replace the resorbed bone volume by an equal volume of new bone.^[^ ^37^, ^38^, ^39^ ^]^ In addition, osteocytes as well as osteoclast and osteoblast progenitors contribute to this coupling, which likely change their contribution during their differentiation toward mature osteoclasts and osteoblasts.^[^ ^40^, ^41^ ^]^ As such, coupling is a highly complex process, and it is expected that not all coupling aspects are present in our model. More specifically, the release of growth factors from the matrix/scaffold and the contribution of osteocytes are likely lacking. To enable coupling through growth factor release, in vitro remodeling models could be developed on decellularized bone tissue.^[^ ^42^, ^43^ ^]^ To additionally involve osteocytes into the bone remodeling process in vitro, a long‐term pre‐culture in which osteoprogenitors differentiate into osteocytes while they develop their mineralized niche,^[^ ^44^, ^45^ ^]^ the 3D embedding and osteocytic differentiation of osteoblasts,^[^ ^46^, ^47^ ^]^ or the use of cell lines might be required, due to challenges with primary osteocyte isolation and subsequent culture.^[^ ^48^ ^]^ To combine the presence of a growth‐factor containing bone matrix and osteocytes, osteocytes could also be cultured in their native niche using human trabecular bone specimens.^[^ ^48^, ^49^ ^]^ In this study we found quantitative coupling between resorption and formation at the tissue level. To further validate coupling in our in vitro model, it would be interesting to study coupling qualitatively at the level of the individual resorption pits to see whether formation takes place on previously resorbed surfaces like in the model of Hikita et al.^[^ ^50^ ^]^ Nevertheless, quantitative coupling was observed along all conditions, indicating some endogenous regulation in all conditions.

The application of mechanical loading tended to be the most influential factor on both resorption and formation outcomes. While mechanical loading in terms of fluid flow induced shear stress has been shown to stimulate mineralization and collagen formation in similar settings, in line with our results,^[^ ^21^, ^44^ ^]^ its clear effect on resorption outcomes was unexpected. In vivo, bone remodeling and adaptation is regulated by osteocytes under influence of interstitial fluid flow through the lacuno‐canalicular network.^[^ ^51^ ^]^ Osteocytes that sense mechanical loading could inhibit osteoclastic differentiation both directly and indirectly.^[^ ^52^ ^]^ The direct influence of mechanical loading on osteoclast differentiation is relatively unknown in vitro with both positive ^[^ ^53^ ^]^ and negative ^[^ ^54^, ^55^ ^]^ influences reported in literature. The contribution of osteocytes and thereby their inhibitory influence on resorption under influence of mechanical loading is likely lacking. Another explanation for the enhanced resorption under influence of mechanical loading could be the improved mass transport when fluid flow was applied. It would therefore be interesting to study the interaction between osteoclastic differentiation factors and mechanical loading within our model, to check whether the likely improved distribution of osteoclastic differentiation factors indeed leads to increased osteoclastic differentiation.

While we believe that the relative simplicity of this model is an advantage for reproducibility and potentially translatability,^[^ ^56^ ^]^ physiological relevance (and thereby complexity) could be increased by the addition of osteocytes,^[^ ^44^ ^]^ vasculature and innervation,^[^ ^57^ ^]^ and potentially other adjacent soft tissues. Also the use of piezoelectric biomaterials could be explored.^[^ ^58^ ^]^ Another limitation of the current setup is that four scaffolds are placed within one bioreactor. Medium analyses therefore only show the average per bioreactor containing two donor combinations. Thereby, we were not able to fully characterize the different cell populations within our highly mineralized model using qPCR, as previously also described elsewhere.^[^ ^59^ ^]^ Another limitation of the current study is the resolution of the fractional factorial design. With the use of a resolution III design, only an influence of factor main effects could be provided. Moreover, these main effects are confounded with interaction effects, which complicates outcome interpretation. Therefore, further studies are required to unravel the potential interactions between factors. In our evaluation, we did not find significant contributions of specific factors to cell viability and bone turnover outcomes. Still clear differences between experimental runs were observed. This suggests that a combination of multiple factors contributed to the found differences between experimental runs, which also means that an untested combination might still be superior. Nevertheless, by both using three different hMC and hMSC donors as well as three different quantitative outcomes for each resorption and formation, we here present a robust evaluation of the impact of culture variables on human bone remodeling in vitro.

## 4 Conclusion

With the aim to mimic physiological balanced bone remodeling in vitro, we have identified the impact of commonly used culture variables on bone turnover parameters in a human bone remodeling model. We herewith present a robust in vitro bone remodeling model, which was able to capture physiological quantitative resorption–formation coupling along all conditions, which could be enhanced by external stimuli. As such, in vitro remodeling (i.e., resorption and formation) was enhanced by the application of mechanical loading. Moreover, high stimulation with osteogenic differentiation factors disturbed mineral resorption–formation coupling. Suitable conditions were found that could be applied to model high bone turnover (run 1—α‐MEM, 1:5, dynamic, 5% hPL, and osteoclast factors) or self‐regulating bone remodeling (run 4—α‐MEM, 1:2, dynamic, 10% hPL, and 1,25‐dihydroxyvitamin D3) as the addition of osteoclastic and osteogenic differentiation factors was not required for remodeling with this combination. Thereby, the results generated with our in vitro model potentially allow for better translation between in vitro studies and toward in vivo studies, for improved preclinical bone remodeling drug development and a mechanistic understanding of human bone remodeling.

## 5 Experimental Section

### Scaffold Fabrication

*Bombyx mori* *L*. silkworm cocoons were degummed by boiling them in 0.2 m Na_2_CO_3_ (S‐7795, Sigma‐Aldrich, Zwijndrecht, The Netherlands) for 1 h. Air‐dried silk fibroin (SF) was dissolved in 9 m LiBr (199870025, Acros, Thermo Fisher Scientific, Breda, The Netherlands), filtered, and dialyzed against ultra‐pure water (UPW) for 36 h using SnakeSkin Dialysis Tubing (molecular weight cutoff: 3.5 K, 11532541, Thermo Fisher Scientific). The dialyzed SF solution was frozen at −80 °C and lyophilized for 7 days. Lyophilized SF was dissolved in hexafluoro‐2‐propanol (003409, Fluorochem, Hadfield, UK) at a concentration of 17% (w/v) and casted in scaffold molds containing NaCl granules with a size of 250–300 µm as template for the pores. Molds were covered to improve the SF blending with the granules. After 3 h, covers were removed from molds, and hexafluoro‐2‐propanol was allowed to evaporate for 7 days whereafter β‐sheets were induced by submerging SF‐salt blocks in 90% MeOH for 30 min. SF‐salt blocks were cut into discs of 3 mm height with a Accutom‐5 (04946133, Struer, Cleveland, OH, USA). NaCl was dissolved for 48 h from the scaffolds in UPW, resulting in porous sponges. From these sponges, scaffolds were punched with a 5 mm diameter biopsy punch.

### Scaffold Mineralization

SF scaffolds were mineralized as previously described,^[^ ^20^ ^]^ using a mineralization solution with 10× SBF ^[^ ^60^ ^]^ and 100 µg mL^−1^ poly‐aspartic acid (pAsp, P3418, Sigma‐Aldrich). Briefly, a 10x SBF stock was prepared. Just prior to mineralization, mineralization solution was prepared by adding 100 µg mL^−1^ pAsp to 10x SBF, followed by the addition of NaHCO_3_ until a final concentration of 10 × 10^−3^ m, both under vigorous stirring. Scaffolds were incubated with 8.6 mL mineralization solution for two weeks at 37 °C on an orbital shaker at 150 RPM in mineralization solution with a solution replenishment after one week. After mineralization, scaffolds were washed 3 × 15 min in an excess of UPW. Scaffolds were sterilized by autoclaving in phosphate buffered saline (PBS) at 121 °C for 20 min.

### hMC Isolation

PBMCs were isolated from human peripheral blood buffy coats (Sanquin, Eindhoven, The Netherlands; collected under their institutional guidelines and with informed consent per Declaration of Helsinki) of three healthy donors. The buffy coats (≈50 mL each) were diluted with 0.6% w/v sodium citrate in PBS (citrate‐PBS) until a final volume of 200 mL and layered per 25 mL on top of 10 mL Lymphoprep (07851, StemCell technologies, Köln, Germany) in 50 mL centrifugal tubes. After density gradient centrifugation (20 min at 800 × *g*, lowest break), PBMCs were collected, resuspended in citrate‐PBS, and washed four times in citrate‐PBS supplemented with 0.01% bovine serum albumin (BSA, 10735086001, Merck KGaA, Darmstadt, Germany). PBMCs were frozen at 10^5^ cells mL^−1^ in freezing medium containing RPMI‐1640 (RPMI, A10491, Thermo Fisher Scientific), 20% fetal bovine serum (FBS, BCBV7611, Sigma‐Aldrich) and 10% dimethyl sulfoxide (DMSO, 1.02952.1000, VWR, Radnor, PA, USA) and stored in liquid nitrogen until further use. Before hMC isolation, PBMCs were thawed, collected in hMC isolation medium containing RPMI, 10% FBS (BCBV7611, Sigma‐Aldrich) and 1% penicillin–streptomycin (p/s, 15070063, Thermo Fisher Scientific), and after centrifugation resuspended in isolation buffer (0.5% w/v BSA in 2 × 10^−3^ m EDTA‐PBS). hMCs were enriched from PBMCs with manual magnetic activated cell separation (MACS) using the Pan Monocyte Isolation Kit (130‐096‐537, Miltenyi Biotec, Leiden, Netherlands) and LS columns (130‐042‐401, Miltenyi Biotec) according to the manufacturer's protocol, and directly used for experiments.

### hMSC Isolation, Expansion, and Seeding

hMSCs were isolated from human bone marrow of three healthy donors (1M‐125, Lonza, Walkersville, MD, USA, collected under their institutional guidelines and with informed consent) and characterized for surface markers and multilineage differentiation, as previously described.^[^ ^61^ ^]^ Bone‐marrow derived hMSCs were frozen at passage 3 or 4 with 5 × 10^6^ cells mL^−1^ in freezing medium containing FBS (BCBV7611, Sigma‐Aldrich) with 10% DMSO and stored in liquid nitrogen until further use. Before experiments, hMSCs were thawed, collected in high glucose DMEM (hg‐DMEM, 41966, Thermo Fisher Scientific), seeded at a density of 2.5 × 10^3^ cells cm^−2^ and expanded in expansion medium containing hg‐DMEM, 10% FBS (BCBV7611, Sigma‐Aldrich), 1% Antibiotic Antimycotic (anti–anti, 15240, Thermo Fisher Scientific), 1% Nonessential amino acids (11140, Thermo Fisher Scientific), and 1 ng mL^−1^ basic fibroblast growth factor (bFGF, 100–18B, PeproTech, London, UK) at 37 °C and 5% CO_2_. After 7–10 days, at around 80% confluence, cells were detached using 0.25% trypsin‐EDTA (25200, Thermo Fisher Scientific) and seeded onto scaffolds at passage 4 or 5.

### hMC‐hMSC Coculture on Mineralized SF Scaffolds

hMSCs were seeded at a density of 0.5 × 10^6^ cells per scaffold and seeding was performed dynamically ^[^ ^62^ ^]^ in 50 mL tubes on an orbital shaker at 150 RPM in expansion medium. After 6 h, scaffolds were transferred to 24‐well plates and hMCs were seeded in hMC isolation medium at a density of 1 × 10^6^ or 2.5 × 10^6^ cells per 20 µL (dependent on the experimental run) by pipetting 20 µL of cell suspension onto the scaffolds. Cells were allowed to attach for 90 min at 37 °C and every 20 min a small droplet of medium from the respective experimental run was added. Per experimental run, four different hMC and hMSC donor combinations (1–3 repeats per donor combination) were seeded on *N* = 8 scaffolds. The cell‐loaded scaffolds were subsequently placed in custom‐made spinner flask bioreactors (two donor combinations on four scaffolds per bioreactor, two bioreactors per experimental run) and cultured statically or dynamically for 28 days at 37 °C and 5% CO_2_ in their respective medium (Tables S1 and S2, Supporting Information). Medium was replaced 3× per week and medium samples were collected on day 2 and weekly from day 7 and stored at −80 °C. Constructs were sacrificed for analyses after 28 days of culture.

### Microcomputed Tomography

On days 2, 7, 14, 21, and 28, scaffolds (*N* = 8 per run) were scanned and later analyzed with a µCT100 imaging system (Scanco Medical, Brüttisellen, Switzerland). Scanning was performed with an energy level of 45 kVp, intensity of 200 µA, integration time of 300 ms and with twofold frame averaging. To reduce part of the noise, a constrained Gaussian filter was applied to all scans with filter support 1 and filter width sigma 0.8 voxel. Follow‐up images were registered to the image of the previous time point, such that voxels at the surface of the scaffold were categorized into resorption site, formation site, or unchanged/quiescent site.^[^ ^63^ ^]^ The scaffold was segmented at a global threshold of 24% of the maximum grayscale value and remodeled scaffold surface was segmented at a global threshold of 7.5% of the maximum grayscale value. This threshold was chosen after registration of cell‐free construct images in such a way that resorption and formation were below ≈1.5% of the total volume to ensure that remodeled volume was caused by the cells instead of by noise. To further reduce noise, only a minimum cluster of two resorbed or formed voxels were included in the analyses. For illustration purposes, day 28 images were also registered to day 2 images for the total resorption and formation visualization.

### PrestoBlue Assay

On day 2, 7, 14, 21, and 28, scaffolds were incubated with 10% v/v PrestoBlue (A13262, Thermo Fisher Scientific) in their respective medium (without supplementation of 1,25‐dihydroxyvitamin D3, osteogenic or osteoclast factors) within their bioreactors for 25 min at 37 °C in the dark. Samples (*N* = 8, four technical repeats from two bioreactors with each four scaffolds per run) were pipetted in duplo in black 96‐well assay plates. Fluorescence (excitation: 530/25 nm, emission 590/35 nm) was measured with a plate reader (Synergy HTX, Biotek). Measured fluorescence was corrected for blank medium samples.

### LDH Activity

On cell supernatants from days 2, 7, 14, 21, and 28, LDH activity was measured (*N* = 8, four technical repeats from two bioreactors with each four scaffolds per run). A 100 µL supernatant sample or NADH (10107735001, Sigma‐Aldrich) standard was in duplo incubated with 100 µL LDH reaction mixture (11644793001, Sigma‐Aldrich) in 96‐well assay plates. Absorbance was measured after 5, 10, and 20 min at 490 nm using a plate reader, and LDH activity was calculated between the 10 and 20 min reactions, using standard curve absorbance values.

### DNA Assay

On day 28, scaffolds (*N* = 4 per run) were washed in PBS, lyophilized, and digested overnight in papain digestion buffer (containing 100 mmol phosphate buffer, 5 mmol l‐cystein, 5 mmol EDTA and 140 µg mL^−1^ papain (P4762, Sigma‐Aldrich)) at 60°C. DNA was quantified using the Qubit Quantification Platform (Invitrogen) with the high sensitivity assay, according to the manufacturer's instructions.

### TRAP Activity

On cell supernatants from days 2, 7, 14, 21, and 28, TRAP activity was quantified (*N* = 8, four technical repeats from two bioreactors with each four scaffolds per run). A 10 µL supernatant sample or p‐nitrophenol standard was in duplicate incubated with 90 µL *p*‐nitrophenyl phosphate buffer (1 mg mL^−1^ *p*‐nitrophenyl phosphate disodium hexahydrate (71768, Sigma‐Aldrich), 0.1 m sodium acetate, 0.1% Triton X‐100 and 30 µL mL^−1^ tartrate solution (3873, Sigma‐Aldrich) in PBS) in 96‐well assay plates for 90 min at 37 °C. To stop the reaction, 100 µL 0.3 m NaOH was added. Absorbance was read at 405 nm using a plate reader and absorbance values were converted to TRAP activity (converted *p*‐nitrophenyl phosphate in nmol mL^−1^ min^−1^) using standard curve absorbance values.

### Cathepsin K Activity

On cell supernatants from days 2, 7, 14, 21, and 28, Cathepsin K activity was quantified (*N* = 8, four technical repeats from two bioreactors with each four scaffolds per run) as described elsewhere.^[^ ^64^ ^]^ A 50 µL supernatant sample or aminomethylcoumarin (A9891, Sigma‐Aldrich) standard was in duplo incubated with 50 µL substrate working solution (100 × 10^−6^ m Z‐LR‐AMC (BML‐P229‐0010, Enzo Life Sciences, Bruxelles, Belgium), 0.1 m sodium acetate trihydrate, 4 × 10^−3^ m EDTA and 4 × 10^−3^ m DTT at pH 5.5 in UPW) in 96‐well assay plates for 30 min at 37 °C. Fluorescence (excitation: 360/40 nm, emission 460/40 nm) was measured with a plate reader and values were converted to Cathepsin K activity (converted Z‐LR‐AMC in µmol mL^−1^ min^−1^) using standard curve fluorescence values.

### ALP Activity

On day 28, scaffolds (*N* = 4 per run) were washed in PBS and disintegrated using two steel balls and a mini‐beadbeater (Biospec, Bartlesville, OK, USA) in 500 µL cell lysis buffer containing 0.2% (v/v) Triton X‐100 and 5 × 10^−3^ m MgCl_2_. ALP activity in cell lysates was determined by adding 20 µL of 0.75 m 2‐amino‐2‐methyl‐1‐propanol (A65182, Sigma‐Aldrich) to 80 µL sample in 96‐well assay plates. Subsequently, 100 µL substrate solution (10 × 10^−3^ m p‐nitrophenyl‐phosphate (71768, Sigma‐Aldrich) in 0.75 m 2‐amino‐2‐methyl‐1‐propanol) was added and wells were incubated at room temperature for 15 min. To stop the reaction, 100 µL 0.2 m NaOH was added. Absorbance was measured with a plate reader at 450 nm and these values were converted to ALP activity (converted p‐nitrophenyl phosphate in µmol mL^−1^ min^−1^) using standard curve absorbance values.

### PICP Quantification

On cell supernatants from days 2, 7, 14, 21, and 28, PICP as collagen formation product was quantified using an enzyme‐linked immunosorbent assay (ELISA, MBS2502579, MyBioSource, San Diego, CA, USA) according to the manufacturer's protocol. Samples (*N* = 2, one sample per bioreactor with each four scaffolds per run) were added in triplicate to antihuman PICP coated microwells. After 90 min incubation at 37 °C, samples were replaced by biotinylated antibody solution followed by 60 min incubation at 37 °C. After thorough washing, HRP‐conjugate solution was added, and plates were incubated for 30 min at 37 °C. Wells were again washed, and substrate reagent was added followed by 15 min incubation in the dark at 37 °C. To stop the reaction, stop solution was added and absorbance was measured at 450 nm in a plate reader. Absorbance values were converted to PICP concentrations using standard curve absorbance values.

### Scanning ElSEM

On day 28, scaffolds (*N* = 1–3 per run) were fixed in 2.5% glutaraldehyde in 0.1 m sodium cacodylate buffer (CB) for 4 h and then washed in CB. Samples were dehydrated with graded ethanol series (37%, 67%, and 96%, 3 × 100%, 15 min each), followed by a hexamethyldisilazane (HDMS)/ethanol series (1:2, 1:1, and 2:1, 3 × 100% HDMS, 15 min each). Samples were coated with 20 nm gold and imaging was performed in high vacuum, at 10 mm working distance, with a 5 kV electron beam (Quanta 600F, FEI, Eindhoven, The Netherlands).

### Histochemical Analysis and Confocal Microscopy

On day 28, scaffolds (*N* = 1–3 per run) were fixed overnight in 3.7% neutral buffered formaldehyde, washed in PBS, permeabilized for 30 min in 0.5% Triton X‐100 in PBS and stained overnight with 1 µmol mL^−1^ CNA35‐OG488 ^[^ ^65^ ^]^ and 0.2 nmol mL^−1^ OsteoSense 680 (NEV10020EX, PerkinElmer, Waltham, MA, USA) at 4 °C to visualize collagen and hydroxyapatite, respectively. After washing with PBS, samples were incubated for 1 h with 1 µg mL^−1^ DAPI and 50 pmol Atto 550‐conjugated Phalloidin (19083, Sigma‐Aldrich). Samples were washed and imaged in PBS images were acquired with a confocal laser scanning microscope (Leica TCS SP8X, 40x/0.95 HC PL APO objective).

### Statistical Analyses

Statistical analyses were performed, and graphs were prepared in GraphPad Prism (version 9.3.0, GraphPad, La Jolla, CA, USA) and R (version 4.1.2) ^[^ ^24^ ^]^ with the Rcmdr DoE plugin (version 0.12‐3, Ulrike Groemping).^[^ ^25^ ^]^ Statistical analyses were only done for day 21 data, as osteoclasts have a limited lifespan of about 14–21 days,^[^ ^66^, ^67^ ^]^ and osteogenesis takes about 14–21 days.^[^ ^68^ ^]^ ALP data was analyzed at day 28, as endpoint analysis. For the comparison between different experimental runs, data were tested for normality in distributions and equal variances using Shapiro–Wilk tests and Levene's tests, respectively. When these assumptions were met, mean ± standard deviation are presented and a one‐way ANOVA was performed followed by Holm–Šídák's post hoc tests with adjusted *p*‐values for multiple comparisons, in which experimental runs were pairwise compared with the negative (run 9) and positive (run 7) control. Other data are presented as median ± interquartile range and were tested for differences with the nonparametric Kruskal–Wallis test with Dunn's post hoc tests with adjusted *p*‐value for pairwise comparisons with the positive and negative control. To quantify the resorption–formation coupling, a spearman correlation coefficient was calculated for the µCT outcome resorbed mineralized volume—formed mineralized volume—and for the supernatant outcome TRAP activity—PICP concentration. As part of the fractional factorial design analysis, factor main effect plots and effect normal plots were prepared for effect visualization and factor significance,^[^ ^25^ ^]^ respectively. Due to failed µCT registration, some experimental runs missed 1–2 out of eight samples for mineral formation and resorption quantification. To allow for a balanced factorial design analysis, the average of the respective experimental runs was included as additional 1–2 sample values. A *p*‐value of <0.05 was considered statistically significant.

## Conflict of Interest

The authors declare no conflict of interest.

## Supporting information

Supporting Information

## Data Availability

The data that support the findings of this study are available from the corresponding author upon reasonable request.
