# Supplementary material for: The Impact of Culture Variables on a 3D Human In Vitro Bone Remodeling Model: A Design of Experiments Approach
Source: Adv Healthc Mater. 2023 Jul 16;12(27):2301205. doi: 10.1002/adhm.202301205 (PMC11469142; doi:10.1002/adhm.202301205)
Supplement: Supplementary file 1 — Supporting Information [file ADHM-12-2301205-s001.pdf]

ADVANCED HEALTHCARE MATERIALS

Supporting Information

for *Adv. Healthcare Mater.*, DOI 10.1002/adhm.202301205

The Impact of Culture Variables on a 3D Human In Vitro Bone Remodeling Model: A Design of Experiments Approach

*Bregje W. M. de Wildt, Lizzy A. B. Cuypers, Esther E. A. Cramer, Annelieke S. Wentzel, Keita Ito and Sandra Hofmann**

Supporting Information

The impact of culture variables on a 3D human in vitro bone remodeling model; a design of experiments approach.

Bregje W.M. de Wildt, Lizzy A.B. Cuypers, Esther E.A. Cramer, Annelieke S. Wentzel, Keita Ito, Sandra Hofmann*

Each experimental run in the fractional factorial design had a unique medium composition (Table S1 and S2).

Table S1. Medium compositions for different experimental runs.

	Base medium	hPL	AA	HEP	L-Glut	Ascorbic acid	β-GP	Dex	RANKL*	M-CSF	vit D3
R1	94% α-MEM	5%	1%	25 mM		50 µg mL^{-1}		10 nM	50 ng mL^{-1}	50 ng mL^{-1}	
R2	94% α-MEM	5%	1%	25 mM		50 µg mL^{-1}	10 mM	100 nM			10 nM
R3	89% DMEM	10%	1%	25 mM	4mM	50 µg mL^{-1}		10 nM			
R4	89% α-MEM	10%	1%	25 mM		50 µg mL^{-1}		10 nM			10 nM
R5	94% DMEM	5%	1%	25 mM	4mM	50 µg mL^{-1}		10 nM	50 ng mL^{-1}	50 ng mL^{-1}	10 nM
R6	94% DMEM	5%	1%	25 mM	4mM	50 µg mL^{-1}	10 mM	100 nM			
R7	89% DMEM	10%	1%	25 mM	4mM	50 µg mL^{-1}	10 mM	100 nM	50 ng mL^{-1}	50 ng mL^{-1}	10 nM
R8	94% α-MEM	10%	1%	25 mM		50 µg mL^{-1}	10 mM	100 nM	50 ng mL^{-1}	50 ng mL^{-1}	
R9	94% α-MEM	5%	1%	25 mM		50 µg mL^{-1}		10 nM			

Abbreviations: run (R), human platelet lysate (hPL), antibiotic antimycotic (AA), HEPES buffer (HEP), L-glutamine (L-glut), β-glycerophosphate (β-GP), dexamethasone (dex), macrophage colony-stimulating factor (M-CSF), receptor activator of nuclear factor κB ligand (RANKL), 1,25-dihydroxyvitamin D3 (vit D3). *Applied from day 2 in culture.

Table S2. Medium components and suppliers.

α-MEM	41061, Thermo Fisher Scientific, Breda, The Netherlands
DMEM	11880, Thermo Fisher Scientific
hPL	PE20612, PL BioScience, Aachen, Germany
Anti-anti	15240, Thermo Fisher Scientific
HEPES buffer	15630, Thermo Fisher Scientific
L-glutamin	X0550-100, Biowest, Nuaillé, France
Ascorbic acid-2-phosphate	A8960, Sigma Aldrich, Zwijndrecht, The Netherlands
β-glycerophosphate	G9422, Sigma-Aldrich
Dexamethasone	D4902, Sigma-Aldrich
RANKL	310-01, PeproTech, London, UK
M-CSF	300-25, PeproTech
1,25-dihydroxyvitamin D3	D1530, Sigma-Aldrich

Abbreviations: human platelet lysate (hPL), antibiotic antimycotic (anti-anti), macrophage colony-stimulating factor (M-CSF), receptor activator of nuclear factor κB ligand (RANKL).

To measure the differences in DNA content between the different experimental runs after 28 days of culture, a DNA assay was performed. No statistically significant differences in DNA content were found.

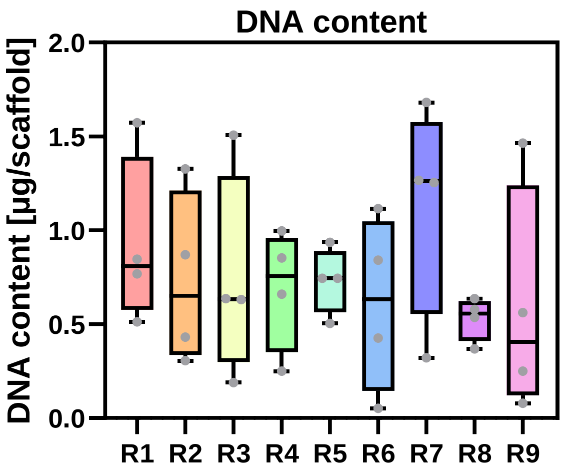

Figure S1. DNA content after 28 days of culture, *ns* (Kruskal-Wallis test),

Normal effect plots were generated to visualize the effect size and direction of each factor on cell viability (Figure S2) and bone turnover outcomes (Figure S3).

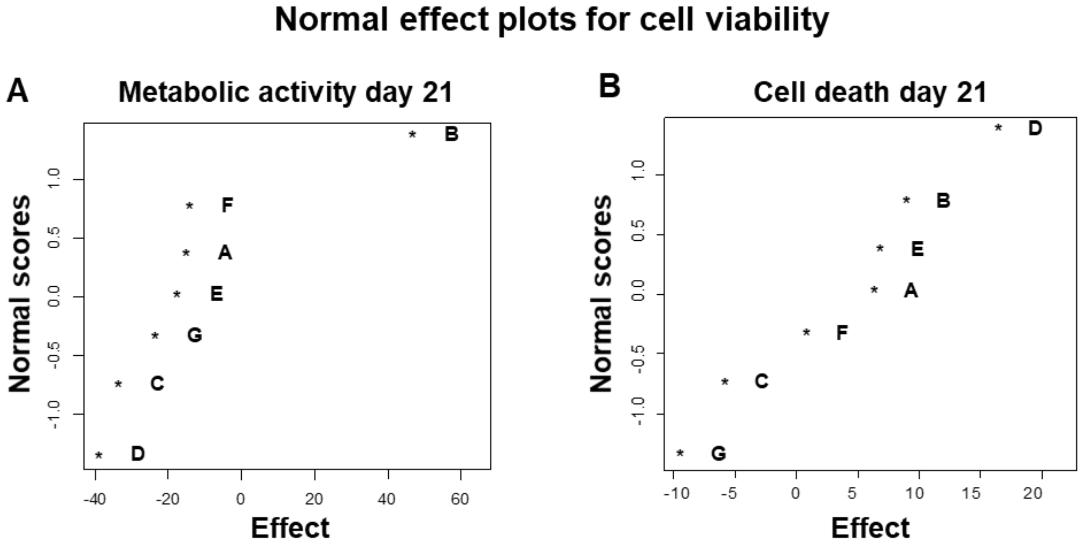

Figure S2. Normal effect plots for cell viability outcomes. (**A**) Normal effect plot for day 21 metabolic activity, indicating a non-significant positive effect of a high cell ratio on metabolic activity. (**B**) Normal effect plot for day 21 cell death. A = base medium, B = cell ratio, C = mechanical loading, D = human platelet lysate concentration, E = osteogenic factors, F = osteoclast factors, G = 1,25-dihydroxyvitamin D3

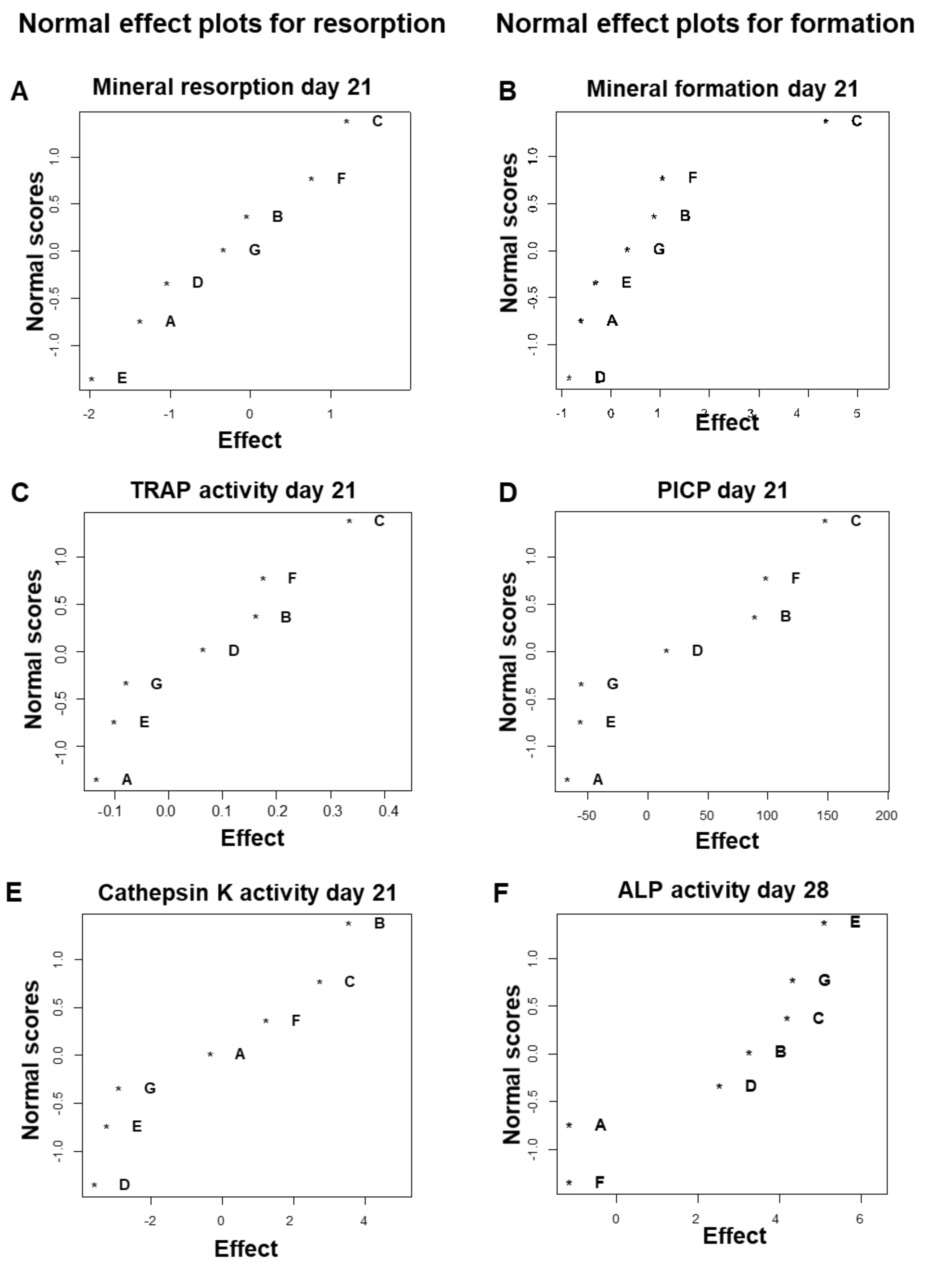

Figure S3. Normal effect plots for bone turnover outcomes. (**A**) Normal effect plot for day 21 mineral resorption. (**B**) Normal effect plot for day 21 mineral formation, indicating a non-significant positive effect of high stimulation with mechanical loading on mineral formation. (**C**) Normal effect plot of day 21 TRAP activity. (**D**) Normal effect plot of day 21 PICP. (**E**) Normal effect plot of day 21 Cathepsin K activity. (**F**) Normal effect plot of day 28 ALP activity. A = base medium, B = cell ratio, C = mechanical loading, D = human platelet lysate concentration, E = osteogenic factors, F = osteoclast factors, G = 1,25-dihydroxyvitamin D3. Abbreviation: tartrate-resistant acid phosphatase (TRAP), pro-collagen 1 c-terminal propeptide (PICP), alkaline phosphatase (ALP).
